# Protective Effects of Pyridoxamine Supplementation in the Early Stages of Diet-Induced Kidney Dysfunction

**DOI:** 10.1155/2017/2682861

**Published:** 2017-10-29

**Authors:** F. Chiazza, A. S. Cento, D. Collotta, D. Nigro, G. Rosa, F. Baratta, V. Bitonto, J. C. Cutrin, M. Aragno, R. Mastrocola, M. Collino

**Affiliations:** ^1^Department of Drug Science and Technology, University of Turin, Turin, Italy; ^2^Department of Clinical and Biological Sciences, University of Turin, Turin, Italy; ^3^Department of Molecular Biotechnology and Health Sciences, University of Turin, Turin, Italy

## Abstract

Pyridoxamine, a structural analog of vitamin B6 that exerts antiglycative effects, has been proposed as supplementary approach in patients with initial diabetic nephropathy. However, the molecular mechanism(s) underlying its protective role has been so far slightly examined. C57Bl/6J mice were fed with a standard diet (SD) or a diet enriched in fat and fructose (HD) for 12 weeks. After 3 weeks, two subgroups of SD and HD mice started pyridoxamine supplementation (150 mg/kg/day) in the drinking water. HD fed mice showed increased body weight and impaired glucose tolerance, whereas pyridoxamine administration significantly improved insulin sensitivity, but not body weight, and reduced diet-induced increase in serum creatinine and urine albumin. Kidney morphology of HD fed mice showed strong vacuolar degeneration and loss of tubule brush border, associated with a drastic increase in both advanced glycation end products (AGEs) and AGEs receptor (RAGE). These effects were significantly counteracted by pyridoxamine, with consequent reduction of the diet-induced overactivation of NF-kB and Rho/ROCK pathways. Overall, the present study demonstrates for the first time that the administration of the antiglycative compound pyridoxamine can reduce the early stages of diet-dependent kidney injury and dysfunction by interfering at many levels with the profibrotic signaling and inflammatory cascades.

## 1. Introduction

One of the most feared chronic microvascular complications of diabetes is diabetic nephropathy. In western world, diabetes is indeed the leading cause of end-stage renal disease, surpassing other etiologies, such as hypertension. One-third of type 1 diabetes mellitus patients and 20% of type 2 diabetes mellitus patients develop end-stage renal disease [1], a pathological condition that dramatically contributes to increasing mortality among diabetic patients if compared to healthy nondiabetic individuals [2].

An early sign of diabetic nephropathy is an increased protein release in urine, displayed as microalbuminuria, which is associated with the progression of renal damage, and is caused by glomerular hypertrophy, hyperfiltration, widening of basement membranes, tubule-interstitial fibrosis, glomerulosclerosis, and podocytopathy [3].

Currently established therapeutic approaches for diabetic nephropathy are primarily antihypertensives that act on renin angiotensin aldosterone system, such as angiotensin-converting enzyme inhibitors and angiotensin II receptor blockers. These appear to reduce proteinuria and to delay, but not prevent, the onset of end-stage renal disease [4].

For this reason, one of the most actual challenging topics is to identify pharmacological treatments that prevent the onset of diabetic nephropathy by acting on the initial stages of diabetes related renal dysfunction. One of the most recently proposed pharmacological tools is pyridoxamine, a structural analog of vitamin B6 that exerts antiglycative effect [5]. Pyridoxamine has been demonstrated to be effective in reducing serum creatinine increase in patients with initial diabetic nephropathy impairment [4, 6]. Pyridoxamine solves its action by inhibiting the formation of advanced glycation end products (AGEs) from glycated proteins and by trapping pathogenic reactive carbonyl compounds (Amadori product), the intermediates in the formation of AGEs [7]. AGEs are irreversible products of protein glycation (glycative stress) expressed ubiquitously and overproduced in case of sugar accumulation, such as during hyperglycemia.

We and others have previously contributed to demonstrating that fructose exposure evokes more AGEs than glucose, mainly because the anomerization equilibrium for fructose is shifted more to the reactive, open chain form of the sugar [8, 9].

Accordingly, accumulation of AGEs (mainly carboxymethyllysine and carboxyethyllysine, CML and CEL) due to chronic exposure to hypercaloric diets significantly contributes to the progression of metabolic, degenerative diseases and related cardiovascular complications [10–17].

Increasing AGEs levels can cause alterations of extracellular proteins, such as collagen and elastin as well as the activation, through the interaction with their receptor RAGE, of different inflammatory signaling pathways in the kidney. Among these, NF-*κ*B is, indeed, the most influent inflammatory signal transduction molecule found to be activated by AGEs-RAGE interaction [18]. Interestingly, recent findings convincingly show that the activation of the proinflammatory NF-kB pathway is also involved in the induction of expression/activity of selective profibrogenetic signaling pathways [19].

However, so far, the potential beneficial effects of pharmacological modulation of AGEs production in the context of diet-related kidney dysfunction have been poorly investigated. To our knowledge, renoprotective effects of pyridoxamine supplementation have been tested only in strains of mice genetically programmed to develop diabetic nephropathy, with limited insights on molecular mechanisms [20–22]. Our study aims to investigate whether oral administration of pyridoxamine prevents the onset of diet-related kidney dysfunction, affecting selective inflammatory and profibrotic signaling pathways in a nongenetic animal model of diet-induced metabolic disease.

## 2. Materials and Methods

### 2.1. Animals and Treatments

Four-week-old male C57Bl/6j mice (Charles River Laboratories, Calco, Italy) were cared for in compliance with the European Council directives (number 86/609/EEC) and with the Principles of Laboratory Animal Care (NIH number 85-23, revised 1985). The scientific project was approved by the Ethical Committee of the Turin University. Mice were randomly allocated into the following dietary regimens: standard diet (SD, *n* = 15) and a high-fat high-fructose diet (HD, *n* = 20) for 12 weeks. Mice were provided with diet and water ad libitum. The HD contained 45% kcal fat (soybean oil and lard), 20% protein (casein), and 35% carbohydrate (fructose) (D03012907 diet, Research Diets). After three weeks of dietary manipulation, two subgroups of SD and HD began pyridoxamine supplementation in the drinking water for the remaining nine weeks (SD + P, *n* = 5; HD + P, *n* = 10). The pyridoxamine dosage (1 g/L, the equivalent of about 150 mg/kg/day) was chosen according to literature data [23] and calculated on the average daily water intake. During the 12 weeks of the experimental protocol, body weight, glycaemia, and food intake were strictly monitored.

### 2.2. Procedures and Plasma Analyses

Six-hour fasting glycaemia was measured at the start of the protocol and every 3 weeks by saphenous vein puncture using a conventional glucometer (GlucoMen LX kit, Menarini Diagnostics). After 12 weeks, mice were anesthetized and euthanized by cardiac exsanguination. Blood was collected and kidneys were rapidly removed and transversely sectioned. One-half was embedded in 4% PAF. The rest of the kidney was frozen in liquid N_2_ and stored at −80°C for protein analysis. Plasma lipid profile and creatinine levels were determined by standard enzymatic procedures using reagent kits. Plasma insulin and urine albumin level were measured using an enzyme-linked immunosorbent assay (ELISA) kit.

### 2.3. Morphological Analysis

Medial sagittal sections of kidney were fixed in 4% formaldehyde solution in M 0.010 phosphate pH 7.4 for 18 h at 4°C. Then, dewaxed 5 *μ*m sections were stained with PAS technique and examined under a light microscope Olympus BX41. Ten randomized pictures at ×200 were obtained to evaluate the degree of tubular damage. Accordingly, with previous publication in which rodents were fed with the same diet [24] tubular damage was expressed in terms of proximal tubules with vacuolar degeneration and loss of the brush border.

In order to evaluate the efficiency of the treatment, administered ad hoc score of damage was applied as follows.

*Grade 1*. There are up to twenty percent of proximal tubules with vacuolar degeneration and disruption of the brush border.

*Grade 2*. There are between twenty-one and forty percent of proximal tubules with vacuolar degeneration and disruption of the brush border.

*Grade 3*. There are more than forty-one percent of the proximal tubules with vacuolar degeneration and disruption of the brush border.

Vacuolar degeneration was defined by the presence of cytoplasmic vacuoles pushing toward the luminal surface of the proximal tubular cell, whereas brush border disruption consisted in a partial or complete loss of its continuity of solution.

### 2.4. Preparation of Tissue Extracts

Kidney extracts were prepared as previously described [25]. Briefly, mice kidneys were homogenized at 10% (w/v) in a Potter Elvehjem homogenizer (Wheaton, NJ, USA) using a homogenization buffer containing 20 mM HEPES, pH 7.9, 1 mM MgCl_2_, 0.5 mM EDTA, 1 mM EGTA, 1 mM dithiothreitol (DTT), 0.5 mM phenylmethyl sulphonyl fluoride (PMSF), and 1 ul/ml PIC. Homogenates were centrifuged at 4000 rpm at 4°C for 5 min and supernatants and pelleted nuclei were separated. Supernatants were removed and centrifuged at 14,000 rpm at 4°C for 40 min. The supernatants thus obtained, containing cytosolic proteins, were carefully removed. The pelleted nuclei were resuspended in extraction buffer containing 20 mM HEPES, pH 7.9, 1 mM MgCl_2_, 0.5 mM EDTA, 1 mM EGTA, 20% glycerol, 420 mM NaCl, 1 mM dithiothreitol (DTT), 0.5 mM phenylmethyl sulphonyl fluoride (PMSF), and 1 ul/ml PIC and centrifuged at 14,000 rpm for 20 min at 4°C. The amount of protein contained in cytosolic and nuclear fractions was determined using a BCA protein assay following the manufacturers' instructions. Samples were stored at −80°C until use.

### 2.5. Western Blotting

Semiquantitative immunoblot analyses of nuclear translocation of p65, expression of CML, CEL, RAGE, vimentin, and fibronectin, and the phosphorylation of IKK*α*/*β*, I*κ*B*α*, RhoA, MYPT1, and Smad2 were carried out in mouse kidney tissue extracts. Equal amounts of proteins were separated by SDS-PAGE and electrotransferred to PVDF membrane. Membranes were incubated with primary antibodies (rabbit anti-NF-kB [1 : 1000], rabbit anti-IKK*α*/*β* [1 : 1000], rabbit anti-Ser^176/180^IKK*α*/*β* [1 : 5000], mouse anti-I*κ*B*α* [1 : 1000], mouse anti-Ser^32/36^I*κ*B*α* [1 : 1000], rabbit anti-RhoA [1 : 1000], rabbit anti-ser^188^RhoA [1 : 1000], rabbit anti-MYPT1 [1 : 1000], rabbit anti-Thr^853^MYPT1 [1 : 1000], rabbit anti-Smad2 [1 : 1000], rabbit anti-Ser^465/467^Smad2 [1 : 1000], rabbit anti-Vimentin [1 : 1000] and rabbit anti-Fibronectin [1 : 5000], goat anti-RAGE, [1 : 1000], mouse anti-CML [1 : 500], and mouse anti-CEL [1 : 100]) followed by incubation with appropriated HRP-conjugated secondary antibodies. Proteins were detected with ECL detection system and quantified by densitometry using analytic software (Quantity-One, Bio-Rad, Hercules, CA, USA). Results were normalized with respect to densitometric value of tubulin for cytosolic proteins or histone H3 for nuclear proteins.

### 2.6. Immunohistochemistry Analysis

RAGE was analyzed by immunohistochemistry on 4 *μ*m formalin-fixed, paraffin-embedded kidney. Slices were deparaffinized in xylol, rehydrated in a graded ethanol series, and subjected to retrieval (20 minutes, 100°C, in tris-EDTA buffer solution, pH 9). Endogenous peroxidase activity was blocked using 0.6% hydrogen peroxide. After blocking, sections were incubated overnight with primary antibody (goat anti-RAGE, 1 : 100, Chemicon International) and subsequently for 1 h with HRP-conjugated anti-goat secondary antibody. Immunohistochemical staining was performed using the Detection System Peroxidase/DAB. Nuclei were counterstained with hematoxylin.

### 2.7. Sirius Red Analysis

Slices were deparaffinized in xylol, rehydrated in a graded ethanol series, and incubated with a saturated aqueous solution of picric acid containing Sirius Red (1 mg/ml) for 1 h. After washing twice in acidified water (containing 0.2% glacial acetic acid), slices were dehydrated in 100% ethanol and cleared with xylol for microscope evaluation.

### 2.8. Statistical Analysis

One-way ANOVA followed by Bonferroni's post hoc test was adopted for comparison among the experimental groups. Data were expressed as mean ± SEM. Statistical tests were performed with GraphPad Prism 6.0 software package (GraphPad Software, San Diego, CA, USA). Threshold for statistical significance was set to *p* < 0.05.

### 2.9. Materials

All compounds were purchased from Sigma Chemical, unless otherwise stated.

## 3. Results

### 3.1. Effects of HD and Pyridoxamine Administration on Metabolic Parameters

Mice fed with the high-fat high-fructose diet (HD) for 12 weeks had greater body weights than control diet-fed littermates (SD) (Table 1). The body weight gain was not significantly reduced by pyridoxamine. The levels of fasting blood glucose and insulin were significantly increased in HD fed mice when compared to SD (Table 1; *p* < 0.05). The concentration of fasting serum glucose, but not insulin, was significantly reduced by daily administration of pyridoxamine (HD + P), remaining still higher than that recorded in the SD group. Chronic exposure to HD strongly increased serum levels of triglycerides and total cholesterol. Most notably, pyridoxamine treatment significantly normalized the changes in triglycerides contents and trendily reduced the levels of total cholesterol.

### 3.2. Effects of HD and Pyridoxamine Administration on Kidney Structure and Function

**Figure 1:**
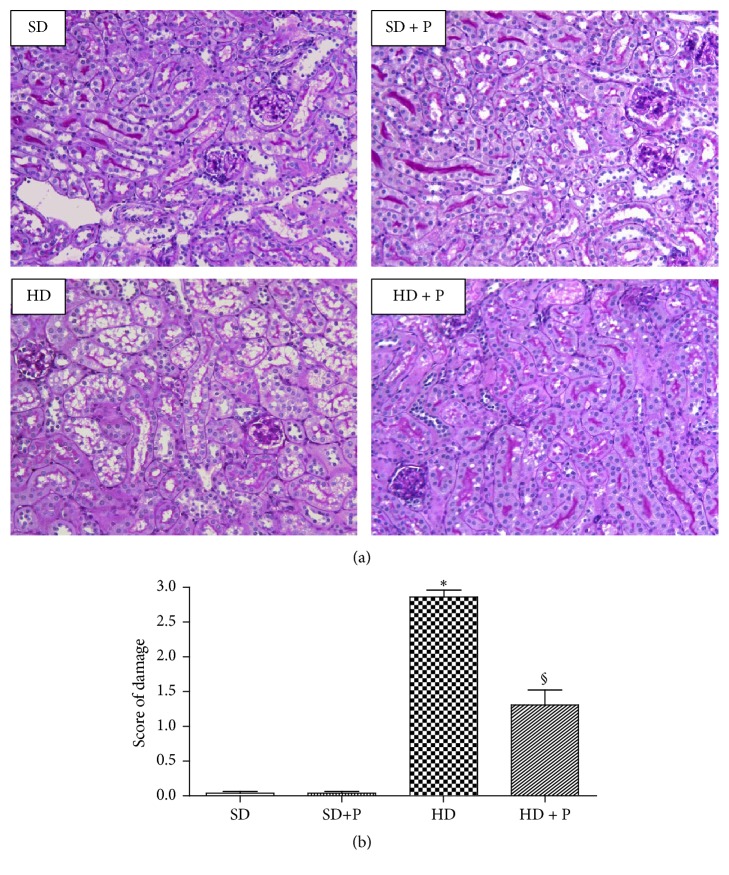
Effects of HD and pyridoxamine administration on kidney structure. (a) Representative kidney section from the experimental group stained with PAS (20x) and (b) score quantification. SD with or without pyridoxamine treatment (150 mg/kg/day): proximal tubules with a normal histoarchitecture. HD: severe degree of tubular vacuolar degeneration and loss of the brush border. HD + pyridox: proximal tubules with almost full protected histology. Values are mean ± SEM of five animals per group. ^*∗*^*p* < 0.05 versus SD; ^§^*p* < 0.05 versus HD.

Representative images from the different experimental groups are shown in Figure 1. Mice fed with the HD exhibited a severe degree of vacuolar degeneration, as well as a complete loss of the brush border integrity. Damage was observed in S1-S2 and S3 parts of the tubule. Pyridoxamine administration prevented the diet-induced morphological alterations. Kidneys from groups fed with chow diet showed proximal tubules with a normal well-preserved histoarchitecture.

**Figure 2:**
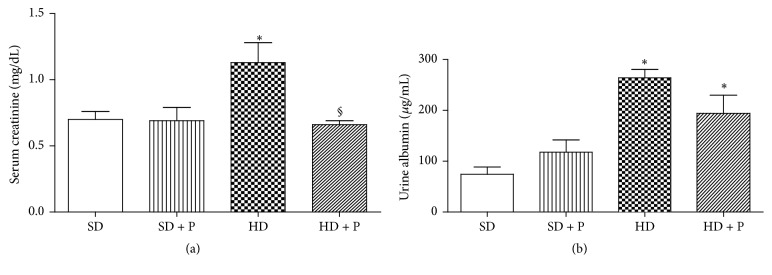
Effects of HD and pyridoxamine administration on kidney function. Serum creatinine (a) and urinary albumin (b) levels were measured in mice exposed to SD or HD in the absence or presence of pyridoxamine (150 mg/kg/day). Values are mean ± SEM of five-ten animals per group. ^*∗*^*p* < 0.05 versus SD; ^§^*p* < 0.05 versus HD.

The HD-induced renal pathology correlated with decline in kidney function, as shown by increased levels of serum creatinine, which was significantly reduced by pyridoxamine administration (Figure 2(a)). Similarly, albumin levels were increased in the HD group compared with the control group and slightly reduced by pyridoxamine treatment, without reaching a statistical significance (Figure 2(b)).

### 3.3. Effects of HD and Pyridoxamine Administration on Kidney AGEs and RAGE

**Figure 3:**
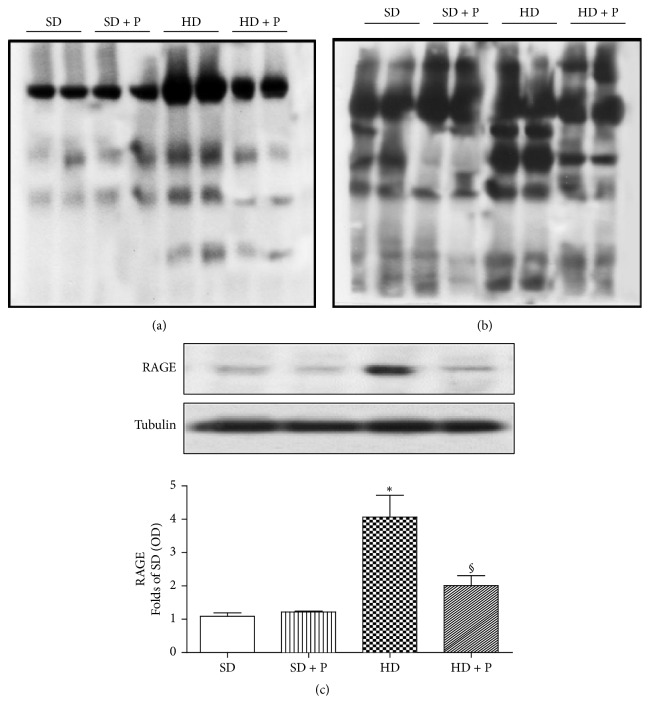
Effects of HD and pyridoxamine administration on kidney AGE and RAGE expression. CEL (a), CML (b), and RAGE (c) were analyzed by western blot on kidney homogenates obtained from mice exposed to SD or HD with or without pyridoxamine administration (150 mg/kg/day). Protein expression is measured as relative optical density (OD), corrected for the corresponding tubulin contents and normalized to the SD band. The data are means ± SEM, five animals per group. ^*∗*^*p* < 0.05 versus SD; ^§^*p* < 0.05 versus HD.

**Figure 4:**
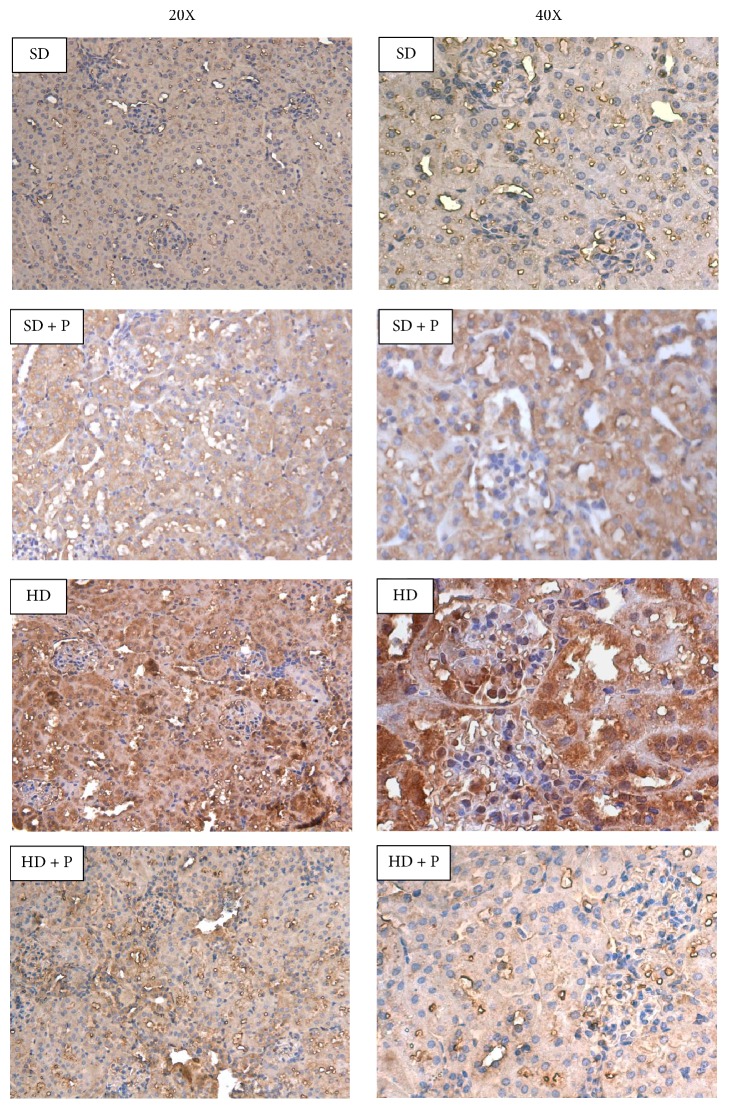
Effects of HD and pyridoxamine administration on kidney RAGE expression. Representative 20x and 40x magnification images of immunohistochemistry analysis for RAGE on kidney sections from mice fed with the SD or the HD, with or without pyridoxamine administration (150 mg/kg/day).

 The causal role of the diet-related activation of the AGEs-RAGE system in evoking activation of proinflammatory and profibrotic pathways, thus contributing to the onset of diet-dependent kidney dysfunction, is well known. Here we measured local expression levels of the most known AGEs carboxymethyllysine/carboxyethyllysine (CML/CEL), showing a massive overproduction at 12 weeks of dietary manipulation (Figures 3(a) and 3(b)). As expected, the daily supplementation with the antiglycative pyridoxamine prevented AGEs accumulation. Immunohistochemistry analysis (Figure 4) demonstrated a parallelism between AGEs overproduction and increased expression of the AGE-receptor RAGE in kidney sections of HD fed mice. This evidence was confirmed and quantified by western blot analysis, showing a threefold increase in RAGE expression in HD group when compared to SD mice (Figure 3(c)). Interestingly, both western blot and immunohistochemical analysis demonstrated that pyridoxamine daily supplementation prevented renal AGEs accumulation as well as RAGE hyperexpression.

### 3.4. Effects of HD and Pyridoxamine Administration on Kidney NF-kB Pathway Activation

**Figure 5:**
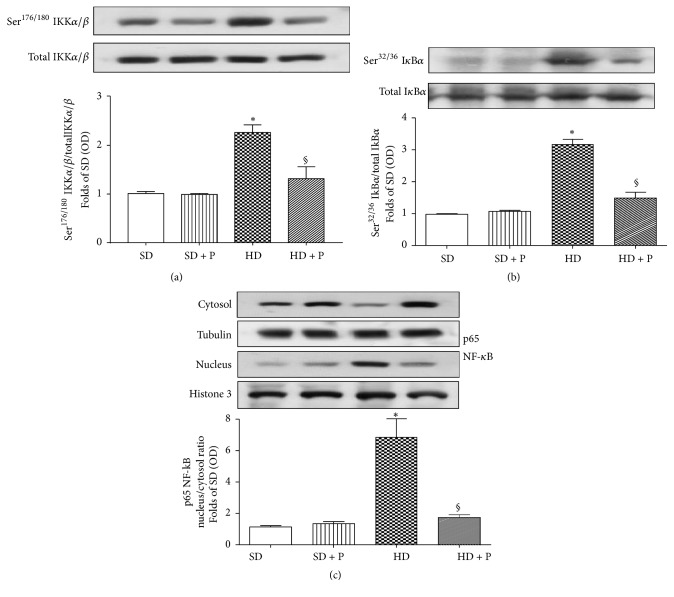
Effects of HD and pyridoxamine administration on kidney NF-kB pathway activation. Total protein expressions of IKK (a) and IkB*α* (b) and their related phosphorylated forms and NF-kB p65 subunit translocation in the nucleus (c) were analyzed by western blot on kidney homogenates obtained from mice exposed to SD or HD, with or without pyridoxamine administration (150 mg/kg/day). Protein expression is measured as relative optical density (OD), corrected for the corresponding tubulin or histone H3 contents and normalized to the SD band. Results are shown as phosphorylated/total protein ratio (a and b) or as nucleus/cytosol protein ratio (c). The data are means ± SEM of three separate experiments, five animals per group. ^*∗*^*p* < 0.05 versus SD; ^§^*p* < 0.05 versus HD.

As shown in Figures 5(a) and 5(b), diet manipulation did not affect total expression of IKK *α*/*β* and I*κ*B*α*, two core elements of the NF-kB signaling cascade. As NF-kB activation mainly occurs via IkB kinase- (IKK-) mediated phosphorylation of the inhibitory molecule, IkB*α*, we measured their levels of phosphorylation showing that HD evoked increased phosphorylation of IKK *α*/*β* on Ser^176/180^ and of I*κ*B*α* on Ser^32/36^ when compared with mice under the SD, suggestive of an increased activation of this pathway.

This overactivation was associated with a marked increased translocation of the p65 NF-*κ*B subunit from the cytosol to the nucleus (Figure 5(c)). In contrast, chronic administration of pyridoxamine to HD fed mice weakened both IKK *α*/*β* and I*κ*B*α* phosphorylation and counteracted HD-induced p65 nuclear translocation (Figures 5(a), 5(b), and 5(c)), thus contributing to reducing local overactivation of a key proinflammatory pathway.

### 3.5. Effects of HD and Pyridoxamine Administration on Kidney Fibrosis

**Figure 6:**
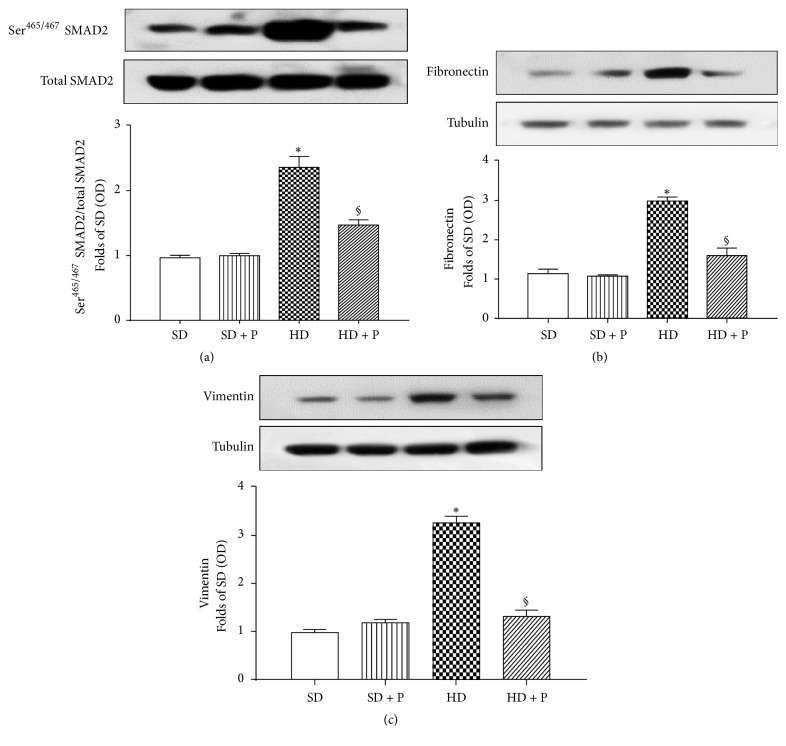
Effects of HD and pyridoxamine administration on profibrotic response. SMAD2 phosphorylation (a) and total protein expression of fibronectin (b) and vimentin (c) were analyzed by western blot on kidney homogenates obtained from mice exposed to SD or HD, with or without pyridoxamine administration (150 mg/kg/day). Protein expression is measured as relative optical density (OD), corrected for the corresponding total SMAD or tubulin contents and normalized to the SD band. The data are means ± SEM, five animals per group. ^*∗*^*p* < 0.05 versus SD; ^§^*p* < 0.05 versus HD.

As shown in Figure 6(a), a robust increase in the phosphorylation of SMAD2 was detectable in the kidney of HD mice. SMAD2 is the canonical profibrotic transcriptional factor that contributes to transmitting TGF-*β* signals from cell surface to the nucleus promoting the production of profibrotic proteins such as vimentin and fibronectin. As expected, the increased activation of SMAD2 was associated with a higher expression of vimentin and fibronectin (Figures 6(b) and 6(c)).

Most notably, pyridoxamine supply significantly reduced the activity of the profibrotic pathway by halving the phosphorylation of SMAD2 as well as the following expression of vimentin and fibronectin.

TGF-*β* also activates the noncanonical pathway of RhoA, a member of the Ras superfamily of small GTP-binding proteins, which contributes to fibronectin production in diabetic kidney.

**Figure 7:**
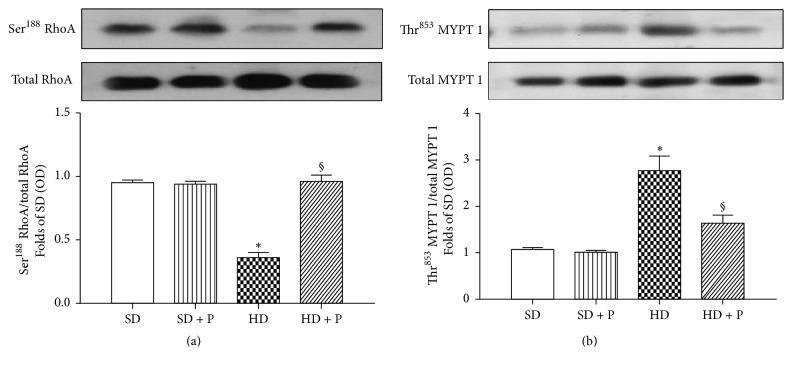
Effects of HD and pyridoxamine administration on kidney RhoA pathway activation. Total protein expressions of RhoA (a), MYPT1 (b), and their related phosphorylated forms were analyzed by western blot on kidney homogenates obtained from mice exposed to SD or HD, with or without pyridoxamine administration (150 mg/kg/day). Protein expression is measured as relative optical density (OD), corrected for the corresponding total protein contents and normalized to the SD band. The data are means ± SEM, five animals per group. ^*∗*^*p* < 0.05 versus SD; ^§^*p* < 0.05 versus HD.

Here we demonstrated that the intensity of RhoA phosphorylation in Ser^188^ was halved in the kidneys of hyper-calorically fed mice when compared to chow fed mice (Figure 7(a)), thus indicating a diet-dependent massive increase of RhoA pathway activity. This effect was associated with a significant increase of the Ser^853^ phosphorylation of MYPT1 (a downstream marker of RhoA/ROCK pathway) (Figure 7(b)). In contrast, treatment of mice exposed to HD with pyridoxamine abolished all the alterations in RhoA/ROCK pathway evoked by HD.

**Figure 8:**
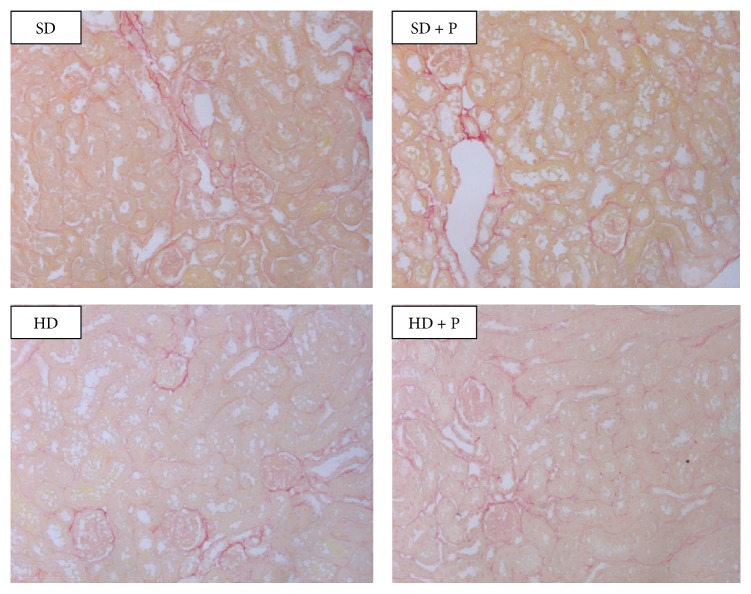
Effects of HD and pyridoxamine administration on kidney fibrosis. Representative kidney section from the experimental group stained with Sirius Red (20x).

However, as shown in Figure 8, no significant difference in the collagen deposition was detectable with Sirius Red assay among the experimental groups, thus demonstrating that a marked fibrotic process was not yet fully developed at 12 weeks of dietary manipulation.

## 4. Discussion

In keeping with previously published papers [24, 26], we confirmed that 12 weeks of dietary manipulation with a high-fat high-fructose diet evoked metabolic derangements, leading to kidney injury, as shown here by alteration in tissue morphology and reduction in its functionality (increased serum creatinine and albuminuria). Most notably, we demonstrated that oral chronic administration of pyridoxamine, a structural analog of vitamin B6 that exerts antiglycative effect, at the dose of 150 mg/kg/die, significantly reduced the metabolic alterations by improving fasting glycaemia and lipid profile. These beneficial effects were associated with improvement in kidney function and morphology due, at least in part, to significant reduction in the local accumulation of CEL and CML (the two most studied AGEs).

In fact, the key role of AGEs accumulation and/or RAGE overexpression in the development of glomerular kidney injury has been recently documented by Yasuhiko and colleagues using transgenic mice overexpressing RAGE in vascular cells. As shown by the authors, RAGE overexpression led to renal dysfunction and advanced glomerulosclerosis and its selective inhibition reverted the renal deleterious effects [27]. Very recently, the involvement of AGEs accumulation in tubular injury and the protective effects of anti-RAGE antibody to block CML-RAGE pathway has been demonstrated in human renal tubular epithelial cell line [28]. According to these findings, the antiglycative compound pyridoxamine has been recently suggested as effective supplementary approach in patients with initial diabetic nephropathy impairment [4, 6]. However, so far, the molecular mechanism(s) underlying its protective role has been slightly examined.

Here we investigated the effects of pyridoxamine on the AGEs/RAGE signaling pathway and the following signaling cascades, focusing on the NF-kB inflammatory signaling pathway. AGEs/RAGE interaction, indeed, stimulates the activation of NF-*κ*B, a key transcriptional factor which connects immune response to infection and inflammation. In its inactive form, NF-*κ*B is sequestered by the inhibitor of *κ*B (I*κ*B) in the cytoplasm and this can be released upon phosphorylation by I*κ*B kinase complex (IKK), which is formed by three subunits termed as IKK*α*, *β*, and *γ*. Once translocated in the nucleus, NF-*κ*B binds to RAGE promoter and further enhances RAGE expression. Thus, the activation of RAGE by AGEs induces RAGE expression [29]. Moreover, NF-kB activation contributes to renal inflammation mostly by upregulating the renal expression of chemokines and cytokines involved in the increasing of vascular permeability such as monocyte chemoattractant protein-1 (MCP-1), IL- (interleukin-) 1, IL-6, IL-18, and TNF (tumour necrosis factor), which are critically involved in kidney disease pathogenesis [19]. Finally, stimulation of RAGE by AGEs increases ROS levels through activation of NAPDH oxidase [30] enhancing levels of oxidative stress and consequently further contributing to an excessive inflammatory response. Our data strongly support the key role of this signaling pathway in the pathogenesis of diet-related kidney dysfunction and the crucial cross-talk mechanism between AGE-RAGE and NF-kB cascades, showing a parallelism between the AGE accumulation/RAGE overexpression and the robust activation and translocation of p65 subunit of NF-kB from the cytosol to the nucleus following dietary manipulation. Interestingly, both the cascades were blunted in their activation by pyridoxamine administration.

Recent findings convincingly showed that the activation of the proinflammatory NF-kB pathway is also involved in the induction of expression and/or activity of selective profibrogenic signaling pathways, including the prosclerotic cytokine TGF-*β* (transforming growth factor-*β*) [19]. Oldfield and others [31] demonstrated that the activation of RAGE induced the overexpression of TGF-*β*, thus demonstrating the involvement of AGEs in the onset of tubulointerstitial fibrosis. Previously published i*n vitro* studies demonstrated that AGEs may evoke production and accumulation of collagen [32, 33].

Unfortunately, we could not confirm these findings, as our model of mild renal injury does not allow the detection of significant accumulation of extracellular collagen. Longer kinetics of dietary manipulation and/or more severe dietary insult would be requested to confirm* in vivo* these findings. However, our main focus was the study of the molecular mechanisms of protection evoked by pyridoxamine, which should be clinically used as supplementary approach to delay the development of diabetic nephropathy and not to counteract a drastic deposition of collagen and renal fibrosis. Thus, we decided to deepen our investigation on potential pharmacological modulation of the two most widely known TGF-*β* depending early profibrotic pathways, whose correlation with AGEs accumulation and pyridoxamine administration has not yet been tested and was not even proposed.

The first cascade is the canonical signaling pathway that involves the phosphorylation and activation of Smad2 and Smad3. Smad4 then binds activated Smad2/3, which enables this complex to translocate to the nucleus and transcribe specific profibrotic genes such as fibronectin and vimentin [34]. Recent studies identified within the inflammation/fibrosis pathway SMAD2/3 an essential modulator of insulin sensitivity related to glomerular dysfunction. Specifically, Smad signaling has an essential role in the development and progression of HFD-induced kidney injury and progression of obesity-related glomerulopathy. Inhibition of Smad signaling, indeed, protects podocytes from metabolic stress through increasing mitochondrial activities [35].

The second cascade is the RhoA/ROCK pathway. RhoA is a member of the Ras superfamily of small GTP-binding proteins, which has been demonstrated to contribute to profibrotic signaling and fibronectin production in diabetic kidney and interfering with VEGF-mediated endothelial cell function [36–38].

Here we demonstrated, for the first time, that both the canonical and noncanonical profibrotic pathways are affected by pharmacological modulation of local AGEs accumulation. In fact, pyridoxamine administration resulted in robust reduction of the enhanced phosphorylation and thus activation of Smad2 and the overexpression of vimentin and fibronectin, two important profibrotic proteins upregulated by Smad2 pathway activation. At the same time, pyridoxamine treatment significantly reverted the activation of the RhoA pathway and the following phosphorylation of MYPT1, a RhoA downstream effector.

These modulatory effects of pyridoxamine on the two fibrotic cascades can be consequences of the reduction of the NF-kB pathway activation due to pyridoxamine interference with the AGEs/RAGE and NF-kB cross-talk mechanism. However, we cannot rule out direct interaction between AGEs and the RhoA/ROCK pathway. In fact, AGEs have been recently demonstrated to directly activate RhoA in endothelial cells, leading to increased hyperpermeability, through a selective complex formation between RAGE and RhoA [39]. Further investigations are needed to better clarify the involvement of NF-kB independent mechanisms of activation of profibrotic pathways by AGEs.

To the best of our knowledge, this is the first study that analyzes from a mechanistic point of view the consequences of pyridoxamine chronic oral administration in a model of diet-induced kidney dysfunction, demonstrating that this antiglycative compound exerts protective beneficial nephrology effects by reducing AGEs levels, leading to interferences with selective inflammatory and profibrotic signaling pathways.

## Figures and Tables

**Table 1:** Effects of HD and pyridoxamine administration on mouse body and tissue weight and blood chemistry at 12 weeks of dietary manipulation. Values are mean ± SEM. ^*∗*^*p* < 0.05 versus SD; ^§^*p* < 0.05 versus HD.

	SD	SD + P	HD	HD + P
	(*n* = 10)	(*n* = 5)	(*n* = 10)	(*n* = 10)
Body weight (g)	25.4 ± 1.3	26.6 ± 3.0	32.8 ± 1.7^*∗*^	34.7 ± 3.6^*∗*^
Food intake (g/day)	2.25 ± 0.07	2.72 ± 0.12	2.30 ± 0.28	2.40 ± 0.14
Kidney weight (% BW)	1.02 ± 0.08	1.08 ± 0.11	0.94 ± 0.10	1.11 ± 0.18
Blood glucose (mg/dl)	72.8 ± 18.9	61.8 ± 16.2	138.8 ± 12.7^*∗*^	104.6 ± 9.7^*∗*§^
Plasma insulin (*μ*g/l)	85.8 ± 5.3	78.1 ± 12.7	106.8 ± 6.8^*∗*^	103.5 ± 11.0^*∗*^
Plasma triglyceride (mg/dl)	37.1 ± 7.6	29.3 ± 5.5	50.5 ± 9.2^*∗*^	34.7 ± 4.7^§^
Plasma cholesterol (mg/dl)	77.2 ± 6.1	84.8 ± 16.4	97.2 ± 8.2^*∗*^	87.2 ± 2.2

## References

[1] Ritz E., Orth S. R. (1999). Nephropathy in patients with type 2 diabetes mellitus. *The New England Journal of Medicine*.

[2] Afkarian M., Sachs M. C., Kestenbaum B. (2013). Kidney disease and increased mortality risk in type 2 diabetes. *Journal of the American Society of Nephrology*.

[3] Gnudi L., Coward R. J., Long D. A. (2016). Diabetic nephropathy: perspective on novel molecular mechanisms. *Trends in Endocrinology & Metabolism*.

[4] Williams M. E., Bolton W. K., Khalifah R. G., Degenhardt T. P., Schotzinger R. J., McGill J. B. (2007). Effects of pyridoxamine in combined phase 2 studies of patients with type 1 and type 2 diabetes and overt nephropathy. *American Journal of Nephrology*.

[5] Khalifah R. G., Baynes J. W., Hudson B. G. (1999). Amadorins: novel post-Amadori inhibitors of advanced glycation reactions. *Biochemical and Biophysical Research Communications*.

[6] Lewis E. J., Greene T., Spitalewiz S. (2012). Pyridorin in type 2 diabetic nephropathy. *Journal of the American Society of Nephrology*.

[7] Turgut F., Bolton W. K. (2010). Potential new therapeutic agents for diabetic kidney disease. *American Journal of Kidney Diseases*.

[8] Mastrocola R., Collino M., Rogazzo M. (2013). Advanced glycation end products promote hepatosteatosis by interfering with SCAP-SREBP pathway in fructose-drinking mice. *American Journal of Physiology-Gastrointestinal and Liver Physiology*.

[9] Suarez G., Rajaram R., Oronsky A. L., Gawinowicz M. A. (1989). Nonenzymatic glycation of bovine serum albumin by fructose (fructation). Comparison with the Maillard reaction initiated by glucose. *The Journal of Biological Chemistry*.

[10] Mastrocola R., Nigro D., Chiazza F. (2016). Fructose-derived advanced glycation end-products drive lipogenesis and skeletal muscle reprogramming via SREBP-1c dysregulation in mice. *Free Radical Biology & Medicine*.

[11] Mastrocola R., Collino M., Nigro D. (2015). Accumulation of advanced glycation end-products and activation of the SCAP/SREBP lipogenetic pathway occur in diet-induced obese mouse skeletal muscle. *PLoS ONE*.

[12] Mastrocola R., Nigro D., Cento A. S., Chiazza F., Collino M., Aragno M. (2016). High-fructose intake as risk factor for neurodegeneration: key role for carboxy methyllysine accumulation in mice hippocampal neurons. *Neurobiology of Disease*.

[13] Schleicher E. D., Wagner E., Nerlich A. G. (1997). Increased accumulation of the glycoxidation product N(epsilon)-(carboxymethyl)lysine in human tissues in diabetes and aging. *The Journal of Clinical Investigation*.

[14] El-Bassossy H. M., Dsokey N., Fahmy A. (2014). Characterization of vascular complications in experimental model of fructose-induced metabolic syndrome. *Toxicology Mechanisms and Methods*.

[15] Lee T.-W., Kao Y.-H., Lee T.-I., Chang C.-J., Lien G.-S., Chen Y.-J. (2014). Calcitriol modulates receptor for advanced glycation end products (RAGE) in diabetic hearts. *International Journal of Cardiology*.

[16] Yang D.-H., Chiang T.-I., Chang I.-C., Lin F.-H., Wei C.-C., Cheng Y.-W. (2014). Increased levels of circulating advanced glycation end-products in menopausal women with osteoporosis. *International Journal of Medical Sciences*.

[17] Daroux M., Prévost G., Maillard-Lefebvre H. (2010). Advanced glycation end-products: implications for diabetic and non-diabetic nephropathies. *Diabetes & Metabolism*.

[18] Ott C., Jacobs K., Haucke E., Navarrete Santos A., Grune T., Simm A. (2014). Role of advanced glycation end products in cellular signaling. *Redox Biology*.

[19] Wada J., Makino H. (2013). Inflammation and the pathogenesis of diabetic nephropathy. *Clinical Science*.

[20] Tanimoto M., Gohda T., Kaneko S. (2007). Effect of pyridoxamine (K-163), an inhibitor of advanced glycation end products, on type 2 diabetic nephropathy in KK-A^y^/Ta mice. *Metabolism—Clinical and Experimental*.

[21] Murakoshi M., Tanimoto M., Gohda T. (2009). Pleiotropic effect of pyridoxamine on diabetic complications via CD36 expression in KK-A^y^/Ta mice. *Diabetes Research and Clinical Practice*.

[22] Abouzed T. K., Munesue S., Harashima A. (2016). Preventive effect of salicylate and pyridoxamine on diabetic nephropathy. *Journal of Diabetes Research*.

[23] Watson A. M. D., Soro-Paavonen A., Sheehy K. (2011). Delayed intervention with AGE inhibitors attenuates the progression of diabetes-accelerated atherosclerosis in diabetic apolipoprotein E knockout mice. *Diabetologia*.

[24] Chiazza F., Couturier-Maillard A., Benetti E. (2015). Targeting the NLRP3 inflammasome to reduce diet-induced metabolic abnormalities in mice. *Molecular Medicine*.

[25] Collino M., Benetti E., Rogazzo M. (2013). Reversal of the deleterious effects of chronic dietary HFCS-55 intake by PPAR-*δ* agonism correlates with impaired NLRP3 inflammasome activation. *Biochemical Pharmacology*.

[26] Mastrocola R., Collino M., Penna C. (2016). Maladaptive modulations of NLRP3 inflammasome and cardioprotective pathways are involved in diet-induced exacerbation of myocardial ischemia/reperfusion injury in mice. *Oxidative Medicine and Cellular Longevity*.

[27] Yamamoto Y., Kato I., Doi T. (2001). Development and prevention of advanced diabetic nephropathy in RAGE-overexpressing mice. *The Journal of Clinical Investigation*.

[28] Yuan Y., Sun H., Sun Z. (2017). Advanced glycation end products (AGEs) increase renal lipid accumulation: a pathogenic factor of diabetic nephropathy (DN). *Lipids in Health and Disease*.

[29] Chavakis T., Bierhaus A., Nawroth P. P. (2004). RAGE (receptor for advanced glycation end products): a central player in the inflammatory response. *Microbes and Infection*.

[30] Wautier M. P., Chappey O., Corda S., Stern D. M., Schmidt A. M., Wautier J. L. (2001). Activation of NADPH oxidase by AGE links oxidant stress to altered gene expression via RAGE. *American Journal of Physiology Endocriology and Metabolism*.

[31] Oldfield M. D., Bach L. A., Forbes J. M. (2001). Advanced glycation end products cause epithelial-myofibroblast transdifferentiation via the receptor for advanced glycation end products (RAGE). *The Journal of Clinical Investigation*.

[32] Iehara N., Takeoka H., Yamada Y., Kita T., Doi T. (1996). Advanced glycation end products modulate transcriptional regulation in mesangial cells. *Kidney International*.

[33] Doi T., Vlassara H., Kirstein M., Yamada Y., Striker G. E., Striker L. J. (1992). Receptor-specific increase in extracellular matrix production in mouse mesangial cells by advanced glycosylation end products is mediated via platelet-derived growth factor. *Proceedings of the National Acadamy of Sciences of the United States of America*.

[34] Meng X., Nikolic-Paterson D. J., Lan H. Y. (2016). TGF-*β*: the master regulator of fibrosis. *Nature Reviews Nephrology*.

[35] Sun Y. B. Y., Qu X., Howard V. (2015). Smad3 deficiency protects mice from obesity-induced podocyte injury that precedes insulin resistance. *Kidney International*.

[36] Kolavennu V., Zeng L., Peng H., Wang Y., Danesh F. R. (2008). Targeting of RhoA/ROCK signaling ameliorates progression of diabetic nephropathy independent of glucose control. *Diabetes*.

[37] Peng F., Wu D., Gao B. (2008). RhoA/rho-kinase contribute to the pathogenesis of diabetic renal disease. *Diabetes*.

[38] Danesh F. R., Sadeghit M. M., Amro N. (2002). 3-Hydroxy-3-methylglutaryl CoA reductase inhibitors prevent high glucose-induced proliferation of mesangial cells via modulation of Rho GTPase/p21 signaling pathway: implications for diabetic nephropathy. *Proceedings of the National Acadamy of Sciences of the United States of America*.

[39] Hirose A., Tanikawa T., Mori H., Okada Y., Tanaka Y. (2010). Advanced glycation end products increase endothelial permeability through the RAGE/Rho signaling pathway. *FEBS Letters*.

